# A novel dendroecological method finds a non-linear relationship between elevation and seasonal growth continuity on an island with trade wind-influenced water availability

**DOI:** 10.1093/aobpla/ply070

**Published:** 2018-11-16

**Authors:** Robert Weigel, Severin D H Irl, Kerstin Treydte, Carl Beierkuhnlein, Johanna Berels, Richard Field, José Carlos Miranda, Alana Steinbauer, Manuel J Steinbauer, Anke Jentsch

**Affiliations:** 1 Experimental Plant Ecology, University of Greifswald, Greifswald, Germany; 2 Disturbance Ecology, Bayreuth Center of Ecology and Environmental Research (BayCEER), University of Bayreuth, Bayreuth, Germany; 3 Biogeography, Bayreuth Center of Ecology and Environmental Research (BayCEER), University of Bayreuth, Bayreuth, Germany; 4 Biogeography and Biodiversity Lab, Institute of Physical Geography, Johann Wolfgang Goethe-University, Frankfurt, Germany; 5 Forest Dynamics, Swiss Federal Research Institute WSL, Birmensdorf, Switzerland; 6 School of Geography, University of Nottingham, Nottingham, UK; 7 GeoZentrum Nordbayern, Department of Geography and Geosciences, Friedrich-Alexander University Erlangen-Nürnberg (FAU), Erlangen, Germany

**Keywords:** Annual density fluctuation, Canary Islands, climatic variability, dendroecology, elevational gradient, endemic, *Pinus canariensis*, seasonality, trade wind clouds, tree rings

## Abstract

Climatic seasonality drives ecosystem processes (e.g. productivity) and influences plant species distribution. However, it is poorly understood how different aspects of seasonality (especially regarding temperature and precipitation) affect growth continuity of trees in climates with low seasonality because seasonality is often only crudely measured. On islands, exceptionally wide elevational species distribution ranges allow the use of tree rings to identify how growth continuity and climate–growth relationships change with elevation. Here, we present a novel dendroecological method to measure stem growth continuity based on annual density fluctuations (ADFs) in tree rings of *Pinus canariensis* to indicate low climatic seasonality. The species ranges from 300 to >2000 m a.s.l. on the trade wind-influenced island of La Palma (Canary Islands), where we measured three decades of tree-ring data of 100 individuals distributed over 10 sites along the entire elevational range. The successfully implemented ADF approach revealed a major shift of stem growth continuity across the elevational gradient. In a remarkably clear pattern, stem growth continuity (percentage of ADFs) showed a hump-shaped relationship with elevation reaching a maximum at around 1000 m a.s.l. Low- to mid-elevation tree growth was positively correlated with the Palmer Drought Severity Index (PDSI; indicating aridity) and sea surface temperature (indicating trade wind-influenced moderation of water supply), while high-elevation tree growth was positively correlated with winter temperature (indicating a cold-induced dormancy period). We conclude that ADFs are a useful method to measure stem growth continuity in low-seasonality climates. Growth of *P. canariensis* on the Canary Islands is more frequently interrupted by winter cold at high elevations and by summer drought at low elevations than in the trade wind-influenced mid elevations, where growth sometimes continues throughout the year. Climate change-associated alterations in trade wind cloud formation might cause non-analogue growth limitations for many unique island species.

## Teaser

Climatic seasonality drives ecosystem processes, but it is poorly understood how different aspects of seasonality affect growth continuity of trees in climates with low seasonality. On islands, exceptionally wide elevational species distribution ranges allow the use of tree rings to identify how growth continuity changes with elevation. Here, we present a novel dendroecological method to measure stem growth continuity based on annual density fluctuations (ADFs) in tree rings of Canary Islands pine on La Palma. We find a remarkably clear pattern that trees most often continue to grow year-round in the humid mid-elevation cloud forests of La Palma.

## Introduction

Climatic seasonality is important for patterns of plant growth as well as for plant distributions. It is thought to affect both productivity (Solantie 2005; Morales *et al.* 2007) and the distribution of plant species (Brown *et al.* 1996; Normand *et al.* 2009). However, evidence for this effect is typically inferred from correlations between productivity or the distribution of species and arbitrary measures of seasonal variability in climate data (e.g. rainfall patterns, Guhathakurta and Saji 2013; continentality index, van der Maaten *et al.* 2017) or normalized difference vegetation index (NDVI) seasonality (e.g. La Cecilia *et al.* 2016). Very simplistic measures are commonly used to capture the strength of climatic seasonality (e.g. range or standard deviation of monthly temperature and precipitation data) from interpolated data that can be of rather poor quality in any given location (Hijmans *et al.* 2005; Fick and Hijmans 2017). While the climatological processes causing spatial patterns of temperature and precipitation seasonality are well understood (Lo Magno *et al.* 2017), it is less clear how spatial differentiation in seasonality affects the actual growing conditions of plants. Thus, the importance of seasonality for ecology and biogeography may be underestimated.

These problems may be especially severe on oceanic islands, where the density of climate stations often does not match the fine-scale spatial heterogeneity of topography and climatic conditions (Fick and Hijmans 2017). Because of the moderating effect of the surrounding ocean, climatic stability—across seasons as well as across years—is considered to be characteristic of subtropical islands when compared with continental areas (Cronk 1997; Weigelt *et al.* 2013). Many islands, however, show pronounced precipitation seasonality that can also vary strongly within an island (Herrera *et al.* 2001; Dewar and Richard 2007; Scholte and Geest 2010), leading to substantial spatial variation in growing conditions for island species. As such, climatic gradients on oceanic islands are based on various interacting drivers of seasonality and can help to understand the ecological relevance of seasonality for plant growth.

Dendroecological research allows the analysis of the effect of climatic seasonality on plant growth by correlating seasonal climate parameters and stem growth of trees (e.g. growth response to summer drought, Battipaglia *et al.* 2014). However, thresholds of climatic seasonality below which year-round growth continuity is induced are not well known. This is mainly because the relationships tend to be species-specific, and result from a complex interaction of climatic conditions, site-specific conditions and timing (Babst *et al.* 2013; Irl *et al.* 2016; Wieser *et al.* 2016). A valuable research opportunity is provided if a single species (a ‘phytometer’) occurs across large gradients of temperature, precipitation and across sites which are assumed to have different climatic seasonality (the degree to which temperature or precipitation amounts change during the course of the year). Since it is impossible to clearly map climatic seasonality due to poor climate data availability on oceanic islands, a ubiquitous phytometer species would allow considerable control and precision in directly assessing growth seasonality (the degree to which periodically unfavourable growing conditions induce a distinctive dormancy period with clear annual growth stops). An excellent example is *Pinus canariensis*, a pine species endemic to the Canary Islands. It is the dominant tree species between 300 and 2000 m elevation on the western island of La Palma (Climent *et al.* 2004; Esteban *et al.* 2005), and therefore occurs under many different climatic and seasonality conditions. *Pinus canariensis* ranges from warm, drought-prone lowlands through humid cloud forests at the trade wind-exposed north-eastern mid elevations of the island, to the cool and dry treeline above the cloud forest and trade wind inversion, where temperature seasonality is increasingly expressed (del Arco Aguilar *et al.* 2010). This natural occurrence of the same tree species, which occasionally continues to grow throughout the year under favourable climatic conditions (Brito *et al.* 2017), along a nearly 2000 m elevational gradient provides an exceptional research opportunity.

Generally, winter cold is associated with abrupt seasonal cessation of cambial growth and formation of clear tree-ring boundaries in temperate regions (Schweingruber 1996; Normand *et al.* 2009). In subtropical tree species, tree-ring analysis is more complex because, on the one hand, intra-annual density fluctuations (IADFs after temporary growth reduction) can appear as false tree rings after summer drought events, in addition to clearly defined tree rings related to winter cold (total growth cessation; Battipaglia *et al.* 2014). On the other hand, subtropical climates with low climatic seasonality, such as on subtropical oceanic islands, are also considered challenging for tree-ring analysis because tree growth may continue throughout the year without formation of distinct tree rings due to the lack of low-temperature thresholds during winter (Cherubini *et al.* 2003). A reduction in cambial activity may then be indicated by density fluctuations of the xylem cells rather than clear tree-ring boundaries (Cherubini *et al.* 2003). Thus, continuity of cambial activity during the whole year might be promoted at low to mid elevations on subtropical oceanic islands, where temperature is relatively stable due to ocean proximity. Towards the upper end of the elevational range, growth seasonality might increase due to higher seasonal temperature control (Normand *et al.* 2009). Beyond such generalizations from continental research, it is poorly known how the seasonal patterns of growth really change along ecological gradients on islands.

Here, we argue that low seasonality in a given location is indicated by a high percentage of years in which growth continues (i.e. does not stop) into the next calendar year. Since from our knowledge no systematic studies exist on these specific wood anatomical growth patterns, here we introduce the term ‘annual density fluctuations’ (ADFs, in analogy to the commonly used term ‘IADFs’, see e.g. Battipaglia *et al.* 2014) that can replace distinct tree-ring boundaries. Annual density fluctuations in *P. canariensis* offer the rare possibility to be utilized as indicators of stem growth continuity—and thus reduced local seasonality—along an extensive elevational gradient. A high percentage of ADFs would indicate continued suitable growth conditions (sufficient water and warmth) and continued cambial activity between years (Micco *et al.* 2012; Battipaglia *et al.* 2014). It is important to separate ADFs, which indicate stem growth continuity between years, from possibly co-occurring IADFs, which indicate growth reductions within one growing season. This separation is obtained by sound tree-ring dating with cross-dating of tree-ring series and correlation of site chronologies to climate station data (Cherubini *et al.* 2003).

Seasonality is a disputed concept, and is often measured very crudely. On islands, the variability of seasonal growth patterns along ecological gradients is poorly known. For ecological purposes, we argue that a more meaningful assessment of seasonality includes the degree to which growth stops in plants. We take advantage of the occasional occurrence of ADFs in tree-ring series of our reference species *P. canariensis*, which grows along an unusually large elevational range. Effectively, we use the percentage of ADFs to measure how stem growth continuity of our reference species changes with elevation on La Palma. We hypothesize that ADFs can indicate continuous annual growth cycles that lack complete growth stops and formation of a tree-ring boundary from one year to the next. This means that ADFs would be tree-ring equivalents for near-continuous growth conditions with mild seasonality (H1). We further hypothesize that in a subtropical island such as La Palma, stem growth continuity is largely driven by a lack of low-temperature thresholds and therefore decreases towards higher elevations because of the greater seasonal variability and amplitude of temperature there compared to more constant temperature nearer the coast (H2). Beyond growth continuity, we also analyse differences in climate–growth relationships across the elevational range of *P. canariensis*. We hypothesize that growth is related to low winter temperatures towards the upper cold and dry treeline, while it is related to summer drought at the drought-prone lower treeline (H3).

## Methods

### Study area

**Figure 1.**
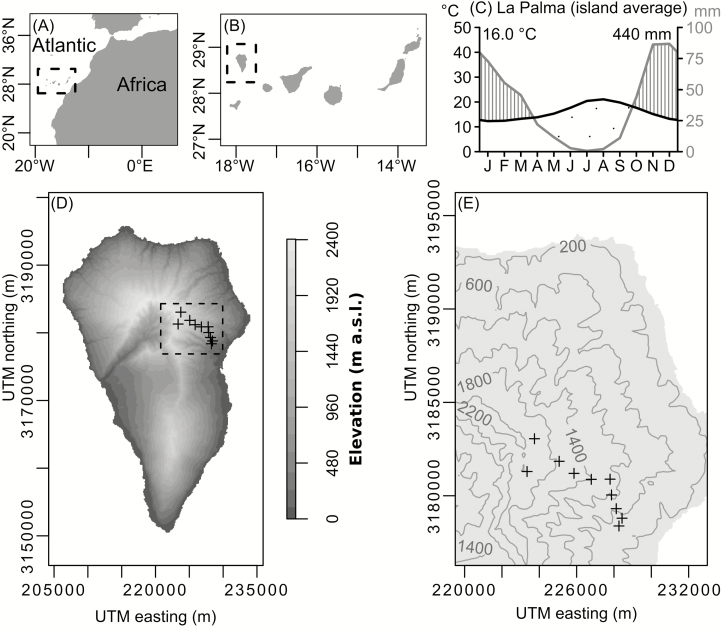
(A) Location of the Canary Islands, Spain; (B) location of La Palma in the north-west of the Canary Archipelago; (C) mean monthly climate variations on La Palma derived from gridded climate data (Fick and Hijmans 2017); (D) location of the study sites on the windward slope of La Palma; (E) topography of the north-eastern part of La Palma. The locations of the study sites of *Pinus canariensis* are marked with ‘+’. Each site includes ≥20 tree cores (two per individual) for cross-validation. Overall sample size is >200 cores.

La Palma is a volcanic oceanic island of 706 km^2^ area and 2426 m a.s.l. maximum elevation in the north-west of the Canary archipelago (Carracedo *et al.* 2002), located off the western coast of Morocco (Fig. 1). The climate of La Palma is subtropical-Mediterranean with mean annual temperatures (averaged over the period 1969–98) varying from low to high elevations, from 20 to 8.7 °C, respectively. August is the warmest month. Annual precipitation mostly occurs in winter (>70 mm/month) with an arid period (down to ≤1 mm/month) from April to September (Fig. 1C). Mean annual precipitation ranges from 170 to almost 1400 mm (Irl *et al.* 2015).

The Azorean high-pressure system, also known as the North Atlantic Subtropical Anticyclone, which is part of the North Atlantic Oscillation (NAO), influences the precipitation on the Canary Islands (Cropper and Hanna 2014). Humid north-eastern trade winds reach the windward slopes, where orographic lifting takes place. A cloud sea is formed when the humid air masses cannot exceed the trade wind inversion layer (TWIL) above 1500 to 1800 m a.s.l., existing most days of the year (Martín *et al.* 2012; Cropper and Hanna 2014). This results in dry conditions on the leeward side of La Palma, while the windward slope is dominated by a non-linear distribution of precipitation along the elevational gradient (Garzón-Machado *et al.* 2014). The humidity maximum is reached within the TWIL, where, besides precipitation, fog water is also available for plants. In the clear air above the TWIL, temperature and especially precipitation decrease sharply but precipitation increases again towards the summit. Below the TWIL, precipitation decreases and meteorological drought increases towards the coast (Herrera *et al.* 2001).

### Study species

**Figure 2.**
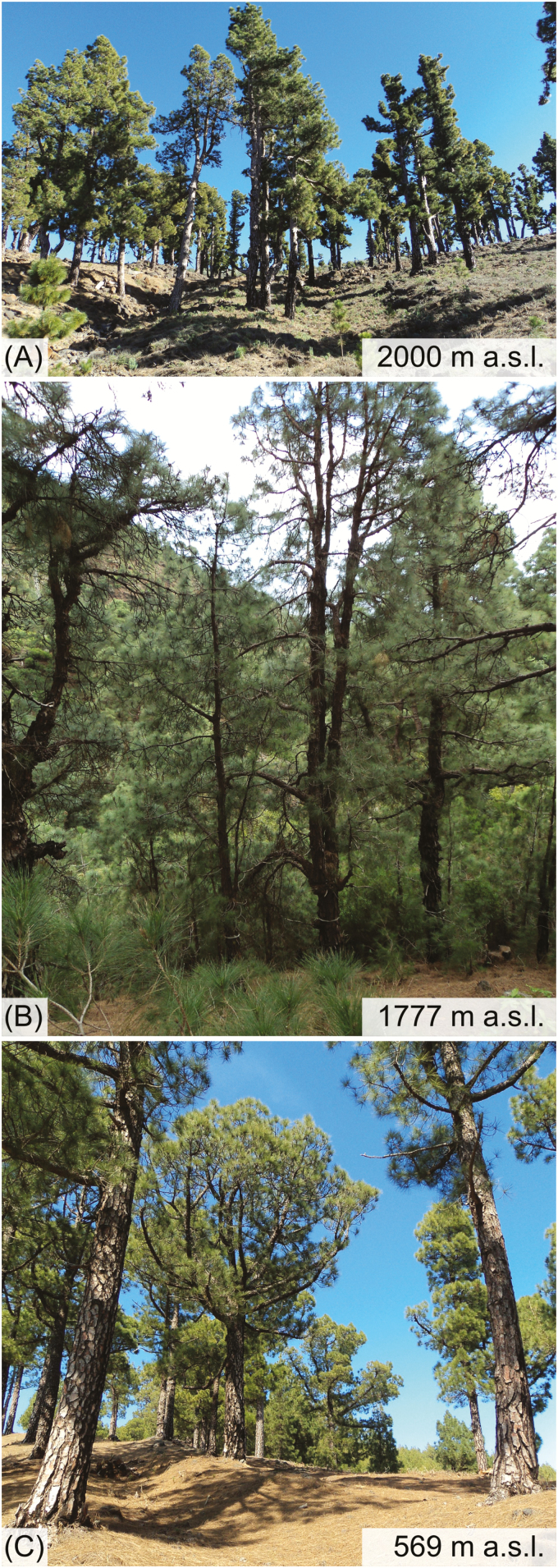
Sampling locations from (A) typical high-elevation, (B) mid-elevation and (C) low-elevation population of *Pinus canariensis* on La Palma.

*Pinus canariensis* is a palaeo-endemic pine species (Pinaceae) of the Canary Islands (Wieser *et al.* 2016) that shows adaptation to a wide range of climatic conditions (López de Heredia *et al.* 2014). *Pinus canariensis* grows up to 60 m high, has long needles (up to 25 cm) enabling it to comb out water from passing clouds, thick bark (up to 7 cm), which can resist fire, serotinous cones allowing a regeneration after fire and it is one of the very few species of Pinaceae which can resprout from epicormic shoots after severe fire (Climent *et al.* 2004). *Pinus canariensis* is widely distributed on the central and western islands of the archipelago from dry, low elevations to the treeline at around 2000 m a.s.l. (Fig. 2; Irl *et al.* 2016; Wieser *et al.* 2016). In the influence zone of the trade winds, laurophyllous species such as *Morella faya*, *Laurus novocanariensis* and the tree heath *Erica arborea* become intermixed and form a subcanopy (del Arco Aguilar *et al.* 2010). Dendrochronologically based studies of *P. canariensis* entail some difficulties because this species is known to cease its cambial activity (resulting in missing rings) not only due to harsh climate conditions (Jonsson *et al.* 2002), but also due to flood damages (Génova *et al.* 2015), volcanic eruptions (Rodríguez Martín *et al.* 2013), human use (extraction of inner heartwood for torch manufacturing, Génova *et al.* 2017) or forest fires (Rozas *et al.* 2011). Since the islands have been populated by humans, forest fires have been a frequent disturbance event of the Canarian ecosystems (de Nascimento *et al.* 2009) on local to regional scale; however, the local fire intensity may strongly vary (Höllermann 2000; Rozas *et al.* 2011; Irl *et al.* 2014). Severe fires may cause considerable growth reductions of *P. canariensis* and temporary decoupling from climatic control, while small to moderate surface fires are only of minor influence on the growth of this fire-adapted tree species (Rozas *et al.* 2011).

### Sampling design, sample processing and ring-width measurements

Ten sites were selected in closed forests along an elevational gradient from 400 to 2100 m a.s.l. on the windward slope (Table 1; Fig. 1C and D). Since our study primarily explores the relationship between stem growth continuity and climatic seasonality, we tried to reduce the influence of non-climatic disturbance events. Therefore, we selected sites where the mature trees did not show obvious signs of previous exposure to severe canopy burning or forest management, such as large fire scars and strong epicormic sprouting (Höllermann 2000; Rozas *et al.* 2011). In addition, the study sites were neither damaged by volcanic eruptions in recent centuries, nor were they located in flooding areas.

**Table 1.** Tree-ring chronology characteristics of *Pinus canariensis* across the elevational gradient. ‘*N*’: number of trees used to analyse climate–growth relationships; ‘EPS’: expressed population signal; ‘IC’: overall inter-series correlation representing the average pairwise correlation between tree-ring series off a site. *n*_missing_ refers to the share of missing rings in all years over all cores of a chronology. The chronologies from 327 to 754 m a.s.l. were removed from subsequent climate–growth analysis due to very low EPS and IC values, which is indicated by italic font.

Elevation (m a.s.l.)	*N*	EPS	IC	*n*_missing_ (%)
2130	7	0.87	0.54	0.5
2000	8	0.76	0.31	0.0
1777	10	0.89	0.47	1.6
1578	8	0.87	0.48	1.3
1379	8	0.87	0.48	0.0
1177	11	0.92	0.53	0.1
1050	9	0.87	0.46	0.0
*754*	*9*	*0.38*	*0.08*	*0.4*
569	8	0.76	0.29	0.8
*327*	*9*	*0.60*	*0.16*	*1.9*

At each site, 10–11 dominant trees were randomly selected within a radius of 50 m. Two cores of each tree were taken with a 5-mm increment corer (Suunto) at breast height, from opposing sides to avoid compression wood (left and right perpendicular to the hillslope). Sampling was done in spring (March) 2014. Tree rings were dated back to 1980 following standard procedures (Stokes and Smiley 1996). This relatively short time window was selected because of logistical limitations. Ring widths were measured with a tree-ring measurement system (LINTAB) and the software TSAP-WIN (Rinntech e.K., Germany) with a resolution of 0.01 mm.

### Identification of density fluctuations

Tree rings were cross-dated within and among trees of a study site visually and with the software COFECHA (Grissino-Mayer 2001) to identify missing tree rings. Furthermore, we also applied the cross-dating procedure as a means to distinguish between ADFs and IADFs because their wood anatomical structure may look similar. We also used marker years as reliable reference to aid correct dating of tree-ring series (Maxwell *et al.* 2011). Those marker years are visually detectable growth anomalies in a tree-ring series. Please note that we did not use the occurrence of ADFs as a reference for cross-dating purposes because some ADFs were not expressed in both cores of a tree. Moreover, proper cross-dating is the basic requirement to distinguish ADFs and IADFs—and not the other way round. A more detailed exploration of within-ring differences of ADFs would require a more detailed analysis of cross sections or sampling of several cores per tree.

**Figure 3.**
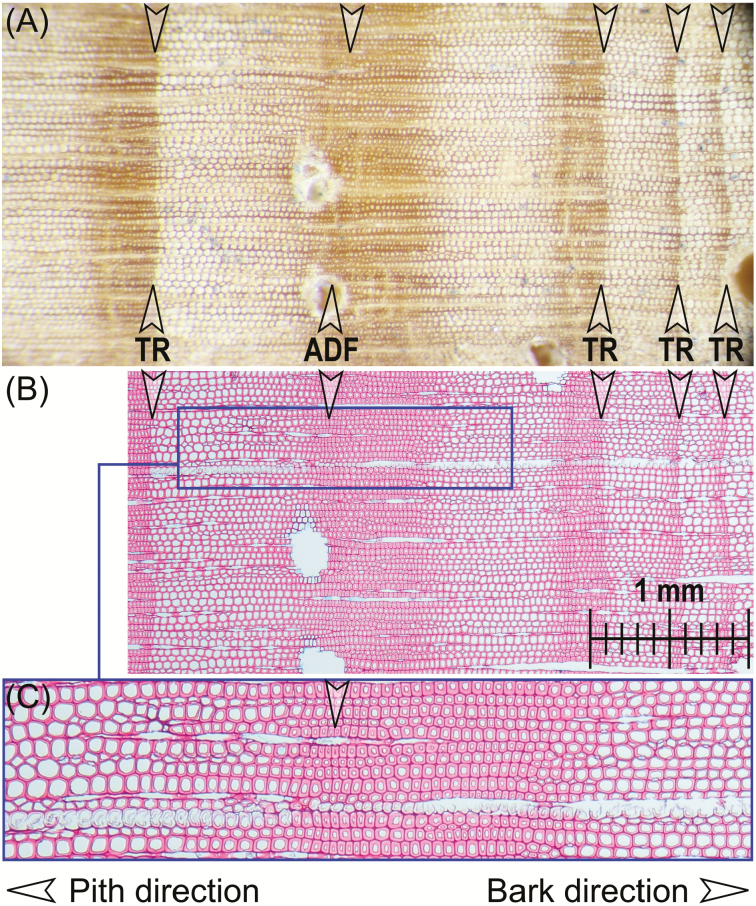
Distinct tree-ring (TR) boundaries versus ADF in wood cores of *Pinus canariensis* from La Palma. (A) Magnified view on a tree core showing an ADF and a neighbouring distinct TR boundary with abrupt stop of latewood formation. The latewood formation of both ADF and TR has comparable dimensions. The ADF is followed by a clearly recognizable TR boundary and two further tree rings with faint latewood boundaries. (B) Thin section showing the same part of the tree core with (C) a magnified view of cell-size transition within an ADF. Arrows indicate the position of smallest late-wood cells where we finished one ring-width measurement and started the next. Images of thin sections by Loïc Schneider.

A tree-ring boundary in gymnosperms is usually characterized by an abrupt transition from small, flattened, thick-walled latewood cells at the end of one growing season to large, thin-walled earlywood cells at the beginning of the next growing season, caused by an abrupt stop in cambial activity (Schweingruber 1996). In contrast, density fluctuations are characterized by a gradual transition of the cell sizes and shape (including earlywood and latewood cells). In our samples, ADFs were distinct from real tree rings due to their specific latewood formation (Fig. 3). The continuous transition from latewood cells of ADFs to earlywood cells of the next growing season clearly contrasts the sharp boundaries of real tree rings. Under certain climatic conditions, radial growth of *P. canariensis* may continue even over consecutive summer and winter seasons of several years (Brito *et al.* 2017). Since these conditions might affect all trees of a site, we were careful about assuming *a priori* that all growth zones that we identified by cross-dating really represent annual growth zones. In particular, we were initially sceptical about exceptionally wide ADFs, which might represent multi-year growth zones. Further, we wanted to test whether we introduced dating errors by confusing IADFs and ADFs systematically in all cores during the cross-dating procedure. *Post hoc*, we therefore verified by correlation with climate data (as described further below) that we indeed identified annual growth zones.

Generally, low climatic seasonality might cause continuous cambial activity (Cherubini *et al.* 2003), resulting in patterns that we here define as ADFs due to low growth seasonality. In tree-ring series with clearly distinguishable annual growth zones, the percentage of ADFs delimiting those growth zones can thus be used as a proxy for stem growth continuity (induced by low seasonality). Therefore, the percentage of ADFs over all considered tree rings was calculated for the last 32 or 33 years, depending on whether the growth period of 2013 had already started or not. Dendrometer measurements indicate that the main growing season of *P. canariensis* may be during March to May (Brito *et al.* 2017). However, dendrometer measurements may primarily represent the dynamics of water uptake by the tree stem and have limitations for recording real xylem cell growth. Wood anatomy studies that would be better suited to assess the seasonal dynamics of xylem cell growth have not been done for *P. canariensis*. Examples from other tree species show that the growing season might be delayed and shortened with increasing elevation (e.g. Moser *et al.* 2010). Accordingly, we recognized during our sampling campaign in March that the main growing season and earlywood cell formation had already started and large earlywood cells had already been formed recently at some of the lower sites. Towards the higher sites, we recognized that a new growing season had not yet started because only small and thick-walled latewood cells formed the outermost part of the xylem. In case of measuring the width of ADF rings, the zone with smallest latewood cells was taken as tree-ring boundary.

### Climatic data

We used interpolated and gap filled monthly climate data from previous July to current December (data access: knmi.nl, Trouet and van Oldenborgh 2013; Climate Research Unit (CRU), Harris *et al.* 2014) for correlation with our tree-ring data. For our observation period 1980–2012, we queried the CRU data for 0.25° × 0.25° gridded temperature, precipitation (Haylock *et al.* 2008) and Palmer Drought Severity Index (PDSI, van der Schrier *et al.* 2013; Osborn *et al.* 2017). Further, we obtained 0.5° × 0.5° gridded sea surface temperature data (SST, Reynolds *et al.* 2007) for the ocean surrounding La Palma. Our study gradient is only represented by a single grid cell in the gridded climate data sets, so that we had to use the same climate time series for correlation with our tree-ring data across sites. We also used high-resolution globally gridded climate data (www.worldclim.org, Fick and Hijmans 2017; resolution 0.5 arcmin; only available as long-term average in this high spatial resolution for the period 1950–2000) to characterize the long-term average temperature and precipitation seasonality at each site. Importantly, this gridded climate data set may rely on climate station data far away from the study area and the gridded precipitation data on high-elevation islands in particular do not adequately reflect the spatial heterogeneity of complex mountain meteorology (Fick and Hijmans 2017). Therefore, the main purpose of showing these interpolated climatic seasonality indicators is to compare their quality to the results of our ADF approach. From the monthly Worldclim data, the temperature seasonality was calculated as the standard deviation of monthly mean temperatures between all 12 calendar months, and precipitation seasonality as the coefficient of variation between all 12 calendar months. We relied on gridded climate data because climate station data for La Palma are scarce, and the precipitation data in particular contain many missing values.

### Statistical data analysis and modelling

To test the relationship between elevation and stem growth continuity (i.e. percentage of ADFs) of all cores, quasi-binomial generalized linear modelling (GLM) was implemented. This allows analysis of change in the percentage of ADFs per tree with elevation, while using the information about how many observations (years) were used to quantify the percentage of ADFs in each tree (in our case most often 33 years). We tested for a linear and quadratic fit of ADFs across elevation, using the Akaike Information Criterion (AIC; Akaike 1974) to select between the two model fits and a null model (no relationship). The modelling was repeated for all cores for each tree side separately to test for effects of pseudo-replication. In addition to AIC based model selection, our results also report *F*-test statistics. Here and in all following analyses, we used a threshold for statistical significance of *P* ≤ 0.05. For quasi-binomial modelling, we state McFadden’s pseudo-*R*^2^ as an indicator of model quality (using the R-package *pscl*).

For the calibration with climate data, all individual tree-ring series, built by averaging two cores per tree, were detrended by using 10-year spline high-pass filters (Cook and Peters 1981) to remove level differences and long-term trends that are potentially unrelated to climate forcing. All climate variables were detrended in the same way. For each site, ring-width chronologies were built from the detrended tree-ring series by calculating bi-weight means. The expressed population signal (EPS; as first defined by Wigley *et al.* 1984 as a theoretical measure on a scale 0–1 of how well the tree-ring chronology of the sample explains the population chronology, Table 1) and the overall inter-series correlation (average pairwise correlation between tree-ring series of a site, measured using Pearson’s correlation coefficient: IC) were calculated for each site (we used the R-package *dplR* (Bunn 2008) for all dendroecological data processing). For later climate–growth relationships, we excluded all sites with very low EPS and IC values (Table 1). In contrast, we did not remove any sites from the ADF analysis because the percentage of ADFs does not rely on temporally explicit information. Subsequently, we correlated (Pearson’s correlation coefficient) monthly climatic variables with the chronologies of all sampled locations and tested for significance (*P* < 0.05) with a 1000-fold bootstrapping procedure (R-package *psych*).

## Results

### Chronology statistics

At almost all sites, a number of individual tree-ring series had to be removed from chronology building due to difficulties in cross-dating (Table 1). Since we had to remove the time series with many missing rings, in particular, the percentage of missing rings in the remaining time series was relatively low (<2 %). All the remaining missing rings occurred only in one core of a tree, so that we could use the opposite core of a tree for identifying the missing ring. Along the whole gradient, the EPS and the mean IC generally increased with elevation. The sites below 800 m a.s.l. and the site at 2000 m a.s.l. had relatively low EPS values (EPS = 0.38–0.76). IC was also relatively low (<0.2) for the sites at 327 and 754 m a.s.l., so we removed those sites from the analysis of climate–growth relationships. Chronology patterns differed between sites. For example, 1995 was a negative marker year (a year with exceptionally small tree rings) at mid-elevation sites **[see****Supporting Information—Fig. S1****]**; other marker years were typically unique to particular sites (e.g. 1997 at 1777 m). Stem growth showed stronger year-to-year variability towards higher elevations, while it was more complacent at sites lower than 800 m a.s.l.

### Stem growth continuity and elevation

The highest stand (2100 m a.s.l.) showed clear annual tree rings and no ADFs during the observation period, while at all other stands at least nine cores contained ADFs. Cores from the stand in the middle of the trade wind zone at 1170 m a.s.l. all showed ADFs. Here, strongly differing between trees, ADFs made up between 20 and >90 % of the annual growth zones per tree. The percentage of ADFs also differed strongly between trees at the lower elevations of the study gradient and here made up between 0 and <90 %.

**Figure 4.**
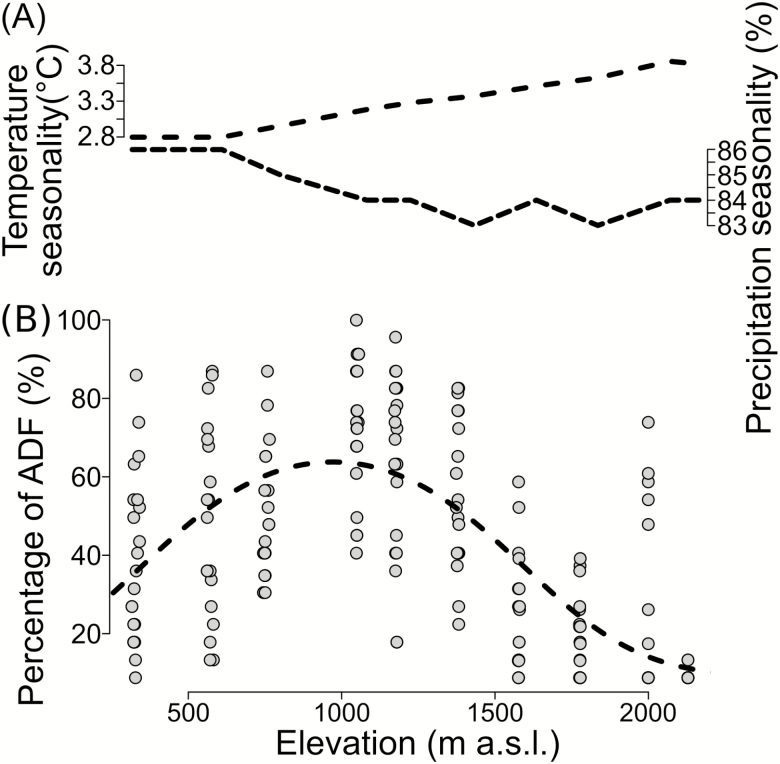
(A) Indices for temperature seasonality (standard deviation of monthly mean temperature) and precipitation seasonality (coefficient of variation of monthly precipitation sum) along the studied elevational gradient derived from gridded climate data (Fick and Hijmans 2017); (B) index for stem growth continuity (percentage of ADFs in the period 1980–2012, definition see text) ranging from 0 (lowest stem growth continuity between years) to 100 % (highest continuity) calculated for all 20 wood cores of *Pinus canariensis* per stand along the elevational gradient. The fitted line represents the quasi-binomial GLM (quadratic fit, *P* < 0.001; pseudo-*R*^2^ = 0.36 on 196 degrees of freedom).

Stem growth continuity (percentage of ADFs per year) thus showed a hump-shaped relationship with elevation (*P* < 0.001; Fig. 4B), reaching a maximum at around 1000 m a.s.l., indicating near-continuous growth throughout the year at mid elevations. From those mid elevations, stem growth continuity decreased towards both the low- and high-elevation treeline of the study gradient. In contrast, climatic seasonality indicated by interpolated climate data (*worldclim*) did not match this hump-shaped relationship. According to the interpolated climate data, precipitation seasonality hardly changed along our studied elevational gradient, but temperature seasonality increased sharply (>35 %) with elevation (Fig. 4A).

### Climatic drivers of tree-ring growth

**Figure 5.**
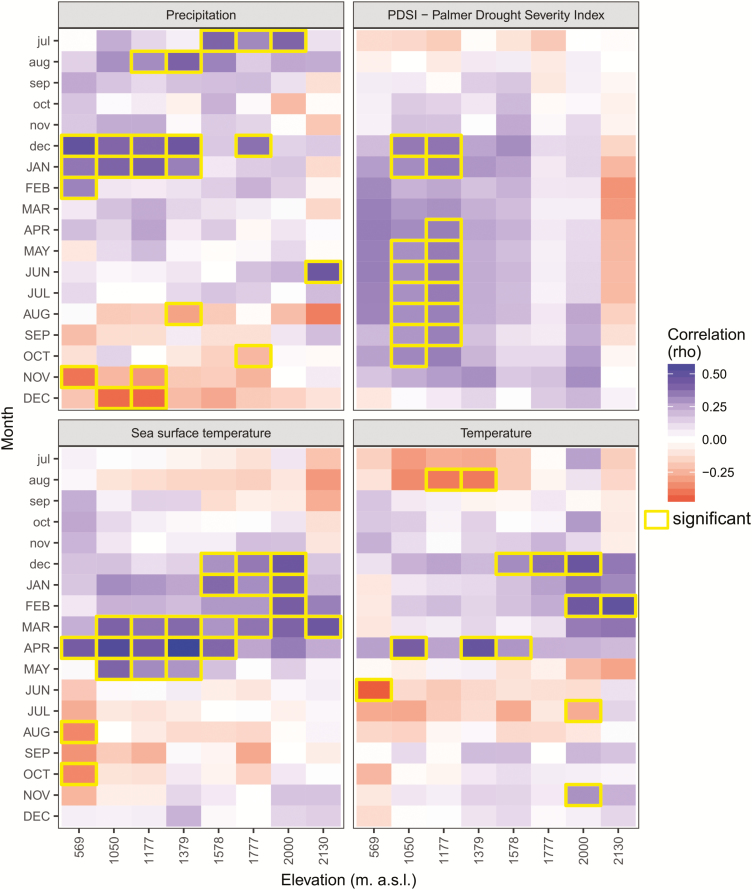
Growth response to monthly climate variables for each study site along the elevation gradient. Climate–growth correlations were calculated for the period 1980–2012 and for the climate variables precipitation, PDSI, sea surface temperature and temperature. Correlations (Pearson’s correlation coefficient) with mean monthly climate variables are shown from previous July (indicated by lower case letters) to current December (upper case letters). Significance (*P* < 0.05) was tested by a 1000-fold bootstrapping procedure.

The statistical relationship between climatic variables and tree-ring growth changed along the elevational gradient (Fig. 5). We could detect some positive correlations of tree growth with precipitation, namely with the previous growing season (previous July and August at mid to high elevations), in the winter prior to the growing season (December and January, at low to mid elevations) and with summer precipitation (June precipitation at the highest sites). Positive correlations of tree growth were found with PDSI during summer months at mid elevations, stretching from previous December to current October. Tree growth correlated significantly (positively) with sea surface temperature of previous winter (December to January) at mid to high elevations and current March to May at almost all sites. However, this signal from March to May faded out towards the higher elevations. For temperature, we found significantly positive correlations of tree growth with previous winter temperatures at 1500–1800 m a.s.l. (December) and at the highest site (February). Growth responded also significantly to the current winter conditions, with negative correlations with November and December precipitation at three sites between 500 and 1200 m a.s.l. We further observed that tree growth correlated significantly (positively) with April temperature at three mid-elevation sites.

## Discussion

In this study, we successfully used the percentage of ADFs to indicate year-round continuous stem growth in our phytometer species *P. canariensis*. By doing this, we assessed climatic seasonality indicated by changes in stem growth continuity along an elevational gradient on the subtropical island of La Palma. Growth was most continuous at mid elevations, probably influenced by the TWIL, where both temperature seasonality and precipitation seasonality are low. Seasonal interruptions in xylem cell production and cambial activity at lower elevations were probably induced by late summer drought and by winter temperature at higher elevations. Thus, we highlight the importance of seasonality measured by ADFs in tree growth in an insular subtropical environment.

### ADFs as a measure for stem growth continuity (H1)

In line with our first hypothesis, we conclude that ADFs in *P. canariensis* are a good indicator of stem growth continuity because both wood density fluctuations and tree-ring boundaries correspond to annual growth zones in the tree-ring series of almost all sites. We differentiated between IADFs and ADFs by cross-dating the tree-ring series within sites. Tree-ring chronologies correlated with annual climate data, which indicates that we did not systematically confuse IADFs and ADFs in certain years (Battipaglia *et al.* 2014).

Even in areas with low climatic seasonality, species often exclusively form tree rings or they exclusively form ADFs (Cherubini *et al.* 2003). However, our results show that *P. canariensis* is very plastic in forming both tree rings and ADFs, even within the same individual. The percentage of ADFs being formed—or not formed at all—by individuals of this species apparently changes along the environmental gradient. Since the occurrence of ADFs and stem growth continuity across seasons are linked (Cherubini *et al.* 2003), the plastic behaviour of *P. canariensis* indicates the usefulness of this tree species as a reference species to measure stem growth continuity with our novel ADF approach. Thus, we suggest ADFs as a robust measure of stem growth continuity, as it is directly connected to the growth pattern of a single tree. We suggest transferring this approach to similarly plastic plant species to explore the manifold species-specific patterns of stem growth continuity along ecological gradients in more detail.

### Elevational pattern of seasonality and stem growth continuity (H2)

In contrast to our second hypothesis, growth appears to be less continuous both at low and high elevations. Indeed, stem growth continuity was highest at mid elevations (800–1200 m a.s.l.), where climatic conditions allow tree growth throughout the year. The decrease of stem growth continuity towards the lower elevations corresponds to an area where water availability is generally reduced and dormancy periods are probably induced by drought late in the growing seasons to avoid cavitation (Fonti *et al.* 2010). The mid elevations with highest stem growth continuity roughly correspond to the lower end of the trade wind zone where fog and precipitation increase water availability, and cloud cover reduces intense solar radiation, evapotranspiration and soil drought stress (Jonsson *et al.* 2002). Thus, our results indicate that influences of climatic seasonality on growing conditions for *P. canariensis* within the mid-elevation cloud forest are buffered—always mild and moist enough for tree growth—throughout the year and that growing conditions there are relatively uniform across years. A decrease of stem growth continuity above 1500 m might be due to environmental conditions that are generally more stressful for tree growth at higher elevation (Liang *et al.* 2010). Here, higher annual temperature amplitude, and particularly low temperatures in winter probably interrupt growth (Normand *et al.* 2009; Fonti *et al.* 2010).

In conclusion, our phytometer approach indicates that continuous growth in *P. canariensis* tends only to happen where both temperature and precipitation are of low variability (mid-elevation cloud forest). Possibly, as soon as either temperature or precipitation seasonality increases (towards higher or lower elevations, respectively), cambial activity stops seasonally and, hence, distinct tree-ring boundaries occur due to dormancy periods. The fact that our ADF approach indicated less stem growth continuity towards higher elevations matches well with the strong elevational increase of temperature seasonality (>35 % increase from lowest to highest elevations) indicated by the interpolated Worldclim gridded climate data. However, the climate data indicated hardly any elevational changes in precipitation seasonality, contrasting the likely drought-induced decrease of stem growth continuity towards lower elevations. This mismatch might be due to the low density of climate stations on the Canary Islands and to the resulting poor quality of gridded precipitation data in particular (Fick and Hijmans 2017) that do not match the fine-scale heterogeneity of precipitation patterns and existence of the cloud forest zone along our studied gradient (Irl *et al.* 2015).

Besides climate, local site conditions along our mountainous study gradient may affect growth continuity, such as moderation of a colder winter microclimate and locally more expressed temperature seasonality due to valley position surrounded by steep slopes (Vitasse *et al.* 2017). Furthermore, stem growth continuity after the main growing season depends on availability of deep soil water reserves and soil aquifer quality (Brito *et al.* 2015, 2017). This may lead to a site-specific or even individual-specific edaphic influence on growth continuity (Leuschner 1996), which is in line with our findings that within-site variance in stem growth continuity strongly increases towards lower elevations where the percentage of ADFs varies considerably between individual trees.

Summing up, local *in situ* growing conditions are only poorly reflected in large-scale climate data; these conditions are better reflected in direct measurements of the physiological growth response. Here, our ADF approach delivered first knowledge on, and general insights into, the spatial patterns of stem growth continuity along elevational gradients on the landscape scale. This should be motivation for conducting more direct physiological measurements of stem growth continuity (e.g. sap flow or dendrometer measurements; Brito *et al.* 2015, 2017; or high-resolution stable isotope analysis; Battipaglia *et al.* 2014; Treydte *et al.* 2014) accompanied by intensive environmental monitoring to deliver detailed insights into the drivers of stem growth continuity on the micro- to local scale for some specific sites.

### Climate–growth relationship (H3)

Climate–growth relationships changed with elevation along our study gradient. In line with our third hypothesis, stem growth (tree-ring width) is mainly related to drought at the lower treeline and to winter cold at the upper treeline. Towards the lower elevations, we found diverse growth patterns. Due to the resulting low EPS and IC, we excluded the sites at 327 and 754 m a.s.l. from analysis of climate–growth relationships. However, we did not exclude the chronology at 569 and 2000 m a.s.l., which had moderately low EPS and IC values. Although the usefulness of EPS to estimate the suitability of a chronology for climate–growth analysis is debated (Buras 2017), climate–growth relationships at these sites should be interpreted with caution. A more robust analysis of climate–growth relationships, particularly at the sites below 1000 m a.s.l., would probably require more extensive sampling at one site (Nehrbass-Ahles *et al.* 2014).

Despite the drought exposure of *P. canariensis* at low elevations, correlations between monthly precipitation in summer and stem growth were weak. This may be due to both the low suitability of gridded climate data to reflect the complex mountain meteorology (Fick and Hijmans 2017) and the water supply for *P. canariensis* from fog drip (Jonsson *et al.* 2002) and deep soil water tapping (Brito *et al.* 2015). Apart from those methodological considerations, our findings along our studied windward elevation gradient are in line with findings from Tenerife by Jonsson *et al.* (2002) and Rozas *et al.* (2013). Although both authors found that *P. canariensis* populations from the dry leeward slopes of the island show a distinct response to summer precipitation, growth of trade wind-exposed populations did not correlate well with monthly precipitation data during the main growing seasons (Jonsson *et al.* 2002; Rozas *et al.* 2013). These windward populations probably have more opportunities to maintain xylem growth during shorter periods of decreased atmospheric water availability (indicated by monthly precipitation data) by tapping deep soil water reservoirs (Brito *et al.* 2015). In contrast to the weak precipitation signal, a positive correlation between growth and PDSI throughout the growing season at the stands below 1200 m a.s.l. points at the growth limitation of *P. canariensis* due to summer drought. Since the calculation of PDSI integrates over several months (Palmer 1965), it is probably better suited to indicating reduced water availability in deep soil water reservoirs after long-lasting soil drying that even affects the stem growth of our windward populations (Jonsson *et al.* 2002; Wang *et al.* 2005; Brito *et al.* 2015).

This dependence on deep soil water reservoirs is also in line with our finding that higher precipitation amounts prior to the main growing season and warm anomalies of sea surface temperature in the early growing season increase stem growth of *P. canariensis*. A warmer sea surface of the surrounding ocean probably means that the circulation of moist air masses from the ocean to the land is boosted, which may feed the deep soil water reservoirs in winter and spring to enhance tree growth (Buckley *et al.* 2007; Brito *et al.* 2015; Chen *et al.* 2015). The correlations with sea surface temperature were stronger at low to mid elevations, indicating that the oceanic influence was more expressed there.

We found that tree growth at elevations between 1000 and 1600 m a.s.l. was enhanced by warm April temperatures. Thus, the thermal optimum of cambial activity of *P. canariensis* seems to be in April at these elevations, which corresponds to dendrometer-based indications that the main growing season of *P. canariensis* is from March to May (Brito *et al.* 2017). During this time, the vegetation could profit from higher temperatures and from higher soil moisture resulting from previous soil water uptake (Brito *et al.* 2015, 2017).

High-elevation ecosystems on (sub)tropical oceanic islands are occasionally exposed to severe ice storms in winter (Irl *et al.* 2012, 2014). The strong influence of these previous winter conditions on plant growth is indicated by the relationship between tree growth and winter temperatures at the high-elevation sites of our study gradient. In addition, the zones above the TWIL on oceanic islands are often claimed to be high-elevation deserts due to a strong decrease in precipitation in combination with high solar radiation (Juan *et al.* 2000). Interestingly, June precipitation only increased growth of *P. canariensis* at our highest study site. This confirms the sensitivity of upper treeline populations from low latitude mountain systems to both winter cold and summer water availability from precipitation (e.g. Esper *et al.* 2007; Cheng *et al.* 2017).

Our study indicates that radial increment of *P. canariensis* can occasionally extend into the next calendar year. This corresponds well with our findings that, very late in the growing season (November–December), tree growth correlates negatively with precipitation (lowest to mid elevations). This may indicate that prolonged or continuous growth of *P. canariensis* may also profit from less cloud cover and higher availability of photoactive radiation late in the year at such sites (Graham *et al.* 2003; Rozas *et al.* 2013).

Although the edaphic control of plant growth might be quite high for immature volcanic soils, the influence is probably negligible along our elevational gradient on the geologically older part of La Palma (Leuschner 1996). Fire disturbance is another non-meteorological factor that influences growth of *P. canariensis* (Rozas *et al.* 2011). Although we avoided choosing sample sites with tree individuals that were previously exposed to strong canopy burning, the distinct growth reductions that we found at mid elevations in 1995 coincide with regionally widespread fire outbreaks on northern La Palma in 1994 (Höllermann 2000). However, these growth reductions also coincide with an exceptionally dry previous winter without sufficient refill of deep soil water reserves (Rozas *et al.* 2011) that might have triggered the growth reduction. Thus, the growth reductions of 1995 clearly show that it is often difficult to identify the ultimate cause of growth patterns of *P. canariensis*, which is exposed to manifold environmental stressors.

## Conclusion

In conclusion, our approach based on dendrochronological assessment of tree-ring formation and density fluctuations (ADFs) in our reference phytometer species, *P. canariensis*, consistently indicated that seasonal interruption of growth as well as bioclimatic differences between years are least in the cloud forest zone—and not close to sea level. Seasonal growth interruptions occurred similarly often at the low- and the high-elevation treelines of *P. canariensis* (though with larger variance at the lower elevation treeline). Thus, the general claim that the proximity to the ocean buffers climatic seasonality and favours continuous growth is rendered too simplistic by our results. The relationship between climatic seasonality and growth continuity along island elevation gradients is rather more complex. Similar unforeseen seasonality patterns are to be expected in other low-seasonality climates. In those climates, our successfully implemented ADF approach, which has proven as a valuable indicator for year-round cambial growth continuity, may generally serve as a suitable seasonality measure. Since the impacts of climate change are uncertain on the Canary Islands, the unique Canarian flora, with its specific adaptations of plant growth to local climate conditions, may be under pressure when exposed to shifting patterns of seasonality in the near future.

## Supporting Information

The following additional information is available in the online version of this article—

**Figure S1**. Detrended tree-ring width (growth signal) of the individual trees (grey curve) and site chronologies (black curves) of all stands from the last 32/33 years as they were used for analysis of climate–growth relationships.

The R-code and data used for the analyses are also available as Supporting Information.

Supplementary Information

Supplementary Figure

Supplementary Material

## Sources of Funding

This publication was funded by the German Research Foundation (DFG) and the University of Bayreuth in the funding programme Open Access Publishing. This publication also contributes to the European H2020 Project 641762 ECOPOTENTIAL: Improving future ecosystem benefits through Earth Observations. We greatly appreciate the support given by the study programme Global Change Ecology (MSc) within the Elite Network of Bavaria.

## Contributions by the Authors

AJ and CB conceived the study. KT designed the study together with CB, JB, AS, AJ, and RW. KT guided the dendroecological analysis from field sampling to lab analysis that was conducted by JB, AS, and RW. MJS and RW conducted the statistical analysis with contributions from JB, JCM, AS, and KT. RW wrote the manuscript together with JB and AS under guidance from SDHI, AJ, and KT. All authors discussed the results and contributed to the manuscript with comments and edits.

## Conflict of Interest

None declared.
