# Supplementary material for: A novel dendroecological method finds a non-linear relationship between elevation and seasonal growth continuity on an island with trade wind-influenced water availability
Source: AoB Plants. 2018 Nov 16;10(6):ply070. doi: 10.1093/aobpla/ply070 (PMC6306105; doi:10.1093/aobpla/ply070)
Supplement: Supplementary Figure [file ply070_suppl_supplementary_figure.pdf]

SUPPORTING INFORMATION

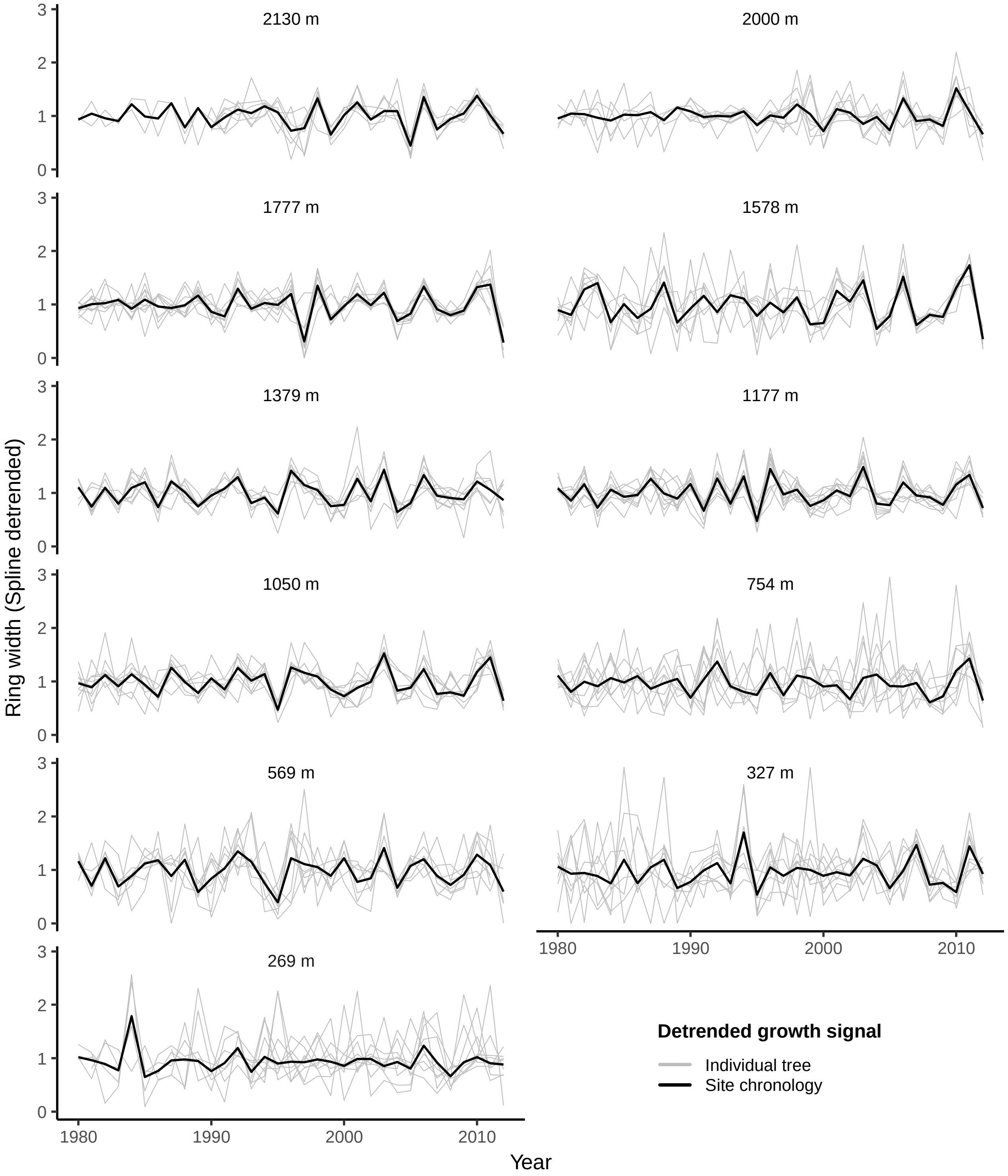

Figure A1. *Detrended tree-ring width (growth signal) of the individual trees (grey curve) and site chronologies (black curves) of all stands from the last 32/33 years as they were used for analysis of climate–growth relationships.*
